# Issues associated with malaria self-medication in sub-Saharan Africa: A systematic literature review and meta-analysis

**DOI:** 10.5281/zenodo.17054133

**Published:** 2025-09-04

**Authors:** John I. Amaka, Ewan MacLeod, Kim Picozzi, Jenna Fyfe, Ifeoma C. Ezenyi, Idayat S. Ijaiya, Daniel D. Attah, Benedict A. Godwin, Victor U. Obisike, Muhammed M. Galamaji

**Affiliations:** 1 Deanery of Biomedical Sciences, College of Medicine and Veterinary Medicine, The University of Edinburgh, Edinburgh, UK.; 2 Current Address: Department of Infectious Diseases, Center for Tropical and Emerging Global Diseases, University of Georgia, Athens, Georgia, USA.; 3 Department of Pharmacology and Toxicology, National Institute for Pharmaceutical Research and Development, Abuja, Nigeria.; 4 Department of Science Education, Waziri Umaru Federal Polytechnic, Birnin-Kebbi, Nigeria.; 5 Department of Animal and Environmental Biology, Kebbi State University of Science and Technology, Aliero, Nigeria.; 6 Department of Science, Waziri Umaru Federal Polytechnic, Birnin-Kebbi, Nigeria.; 7 Department of Public Health, Abia State University, Uturu, Nigeria.; 8 Department of Biology, Federal University of Agriculture, Makurdi, Nigeria.

## Abstract

**Background:**

Malaria is a leading cause of death in sub-Saharan Africa, which is home to more than 90% of both cases and deaths globally. Self-medication with antimalarials is a common practice in the region, mainly due to high malaria endemicity, poverty, and difficulty in accessing services in formal settings. Malaria self-medication is implicated in the rising trend of antimalarial drug resistance which threatens decades of gains made in controlling the disease. Previous studies have somewhat itemised the reasons for malaria self-medication and the factors driving it but have not been able to estimate the overall prevalence of the practice and its dynamics over time regarding period, region and country.

**Materials and Methods:**

Following the Preferred Reporting Items for Systematic Reviews and Meta-Analyses (PRISMA), a systematic review of literature and meta-analysis on malaria self-medication in sub-Saharan Africa was conducted by searching PubMed, Cochrane Library, Web of Science, Scopus and Embase databases for relevant studies written in English and published up to 12th April, 2023, using a combination of different keywords derived from the main keywords (‘malaria’, ’self-medication’ and ‘sub-Saharan Africa’), broadening chances of retrieval by using Boolean operators ‘OR' and ‘AND’.

**Results:**

Twenty-seven studies met the inclusion criteria and were included in the review, giving rise to a pooled prevalence of 55.3% for malaria self-medication. Factors driving self-medication with antimalarials in the region include low-income level, cheap availability of non-prescription drugs, large family size, lack of health insurance, difficulty in accessing healthcare in formal settings and previous satisfactory use of specific drugs.

**Conclusion:**

Due to the underlying factors driving the practice, health authorities and regulatory agencies in sub-Saharan Africa should step up actions by incorporating stakeholders in the informal drug market into a framework that advocates for an enlightened use of antimalarial drugs in the management of the disease.

## Introduction

Malaria continues to threaten nearly half of the world’s population, in 87 countries and territories, especially in low and middle income countries (LIMCs) where children and pregnant women are most affected [1]. Out of the estimated 263 million cases and 597,000 deaths due to malaria globally in 2023, sub-Saharan Africa carries a disproportionate high share of the burden of about 95% of cases and 96% of deaths [1-4], with children <5 yrs accounting for 80% of deaths [3,4].

Antimalarial drug resistance, which was first described in the late 1950s in the Greater Mekong subregion and more recently in Africa, has become a huge threat to global malaria control efforts in the last decade [4,5]. It is presumed that drug resistance in *Plasmodium* is associated with irrational use of anti-malarial drugs including self-medication [6,7]. Self-medication with antimalarials is common in sub-Saharan Africa mainly due to high malaria endemicity, personal convenience and high cost of accessing health services in formal settings [7,8].

One of the greatest threats to malaria control efforts is the emerging issue of antimalarial drug resistance which has been confirmed in two of the human *Plasmodium* species (*P. falciparum* and *P. vi-vax*) [9]. Chloroquine resistance first occurred in late 1950s and early 1960s in three areas of South-east Asia, Oceania, and South America, spreading later to other regions of the world, notably sub-Saharan Africa, where falciparum malaria is transmitted [9]. *P. falciparum* has also developed resistance to currently used drugs including sulfadoxine/pyrimethamine, mefloquine, halofantrine, quinine, and most recently artemisinin [9].

Artemisinin resistance has been confirmed in five countries in southeast Asia [10,11], and some parts of sub-Saharan Africa [11]. Exposure of *Plasmodium* to sub-therapeutic doses of artemisinin over a long period of time and the availability of substandard forms of the drug have been the major driving forces behind the selection of resistant strains of the parasite [12].

Counterfeit and substandard antimalarial drugs also play a huge role in the development of resistance of *Plasmodium* to antimalarial drugs. Fake and substandard antimalarial drugs, which contain little or no active ingredients, can prolong illness and increase the risk of severe disease or death, and if widely used can lead to development of drug-resistant parasites [13]. Counterfeit and substandard antimalarial drugs are common in developing countries where there are weak drug regulatory frameworks/agencies as well as lack of resources required for the effective evaluation of drug quality [13]. An estimated one-third of antimalarial drugs circulating in sub-Saharan Africa failed chemical analysis, packaging analysis or were falsified [14].

The practice of self-medication is a huge threat to public health especially in developing countries where its prevalence is significantly higher due to factors such as poverty and inaccessibility of services in formal health settings [15]. Self-medication raises policy issues that are particularly problematic, one of which is regulation of drugs [16]. One in ten drugs in developing countries is either of poor quality or falsified [17]. The situation in sub-Saharan Africa highlights a lack of, or weak enforcement of, regulation of importation activities of antimalarial drugs, with minimal involvement of the private sector in the training and planning of delivery of services as well as dissemination of knowledge on the correct treatment regimens [18]. These could provide a fertile ground for incidence of antimalarial drug resistance and treatment failure.

As most malaria cases occur in areas where healthcare systems are poorly funded and a large majority of the population has difficulty accessing services in formal settings [16], the Global Partnership Roll Back Malaria Summit in Abuja in 2000 recognised this problem and called upon member states to make treatment of malaria widely available, including for home treatment [19].

In many LMICs, self-medication is an important resource for the management of malaria, with a resort to formal healthcare services only when the situation becomes severe [20]. However, self-medication with antimalarials is particularly concerning as it is associated with sub-therapeutic drug pressure on circulating parasite populations which fuels antimalarial drug resistance [16, 21-22]. Pregnant women are particularly at risk as self-medication could control fever without necessarily controlling parasitaemia, thereby increasing the risk for placental and/or complicated malaria [21]. There is also the tendency to discontinue treatment especially among pregnant women due to non-recognition of symptoms or perceived ineffectiveness of antimalarial drugs [23].

Standby emergency treatment (SBET), which is a strategy for travellers in low or moderate malaria risk areas whereby they are equipped with emergency malaria treatment for self-administration when no formal healthcare services are available, is gaining momentum against the backdrop of global proliferation of counterfeit antimalarial drugs, a situation that is prevalent in Asia where more than 50% of artemisinin products are fake, and is on the rise in sub-Saharan Africa [24]. First recommended to Swiss travellers visiting Thailand in 1988, there is little evidence on its effectiveness as there are arguments about the wasted resources of unused antimalarial drugs [25].

Malaria self-medication should not be confused with home-based management of malaria (HMM). The latter is a strategy developed by WHO to reduce malaria burden in endemic populations by making use of community health workers to educate caregivers so they can provide prompt treatment to suspected cases of the disease in their various homes [26]. The reasons for self-medication with antimalarials include lack of knowledge on the dangers of self-medication, easy access to drugs and self-diagnosis based on presumptive signs and symptoms of malaria [27].

Studies have indicated that inappropriate self-medication results in conditions such as antimicrobial resistance (AMR), adverse reactions, disease masking, drug interactions, misdiagnosis of disease, increased morbidity and wastage of healthcare resources [28]. In many malaria-endemic countries, there is widespread poverty, and healthcare is poorly funded, resulting in most of the population finding it difficult to access services in formal settings, and resorting to self-medication [8,16]. With the emerging evidence of artemisinin resistance in Africa [11, 29-30], there could be treatment failures if issues associated with the irrational use of antimalarial drugs including self-medication in the region are not categorised and addressed. This study, therefore, aimed not only to determine the overall prevalence of malaria self-medication in sub-Saharan Africa but also to identify and categorise various factors associated with malaria self-medication with a view to making appropriate recommendations on the way forward.

The objectives of this study were to: Itemise the reasons for malaria self-medication in sub-Saharan Africa; Identify medications or any other type of treatment used for malaria self-medication in the region; Determine the prevalence of malaria self-malaria self-medication in sub-Saharan Africa? What are the sources of drugs used for malaria self-medication in sub-Saharan Africa? What is the prevalence of malaria self-medication in sub-Saharan Africa? What are the factors that drive malaria self-medication in sub-Saharan Africa?

## Materials And Methods

A systematic search of literature published up to 12 April 2023 was conducted in different databases (PubMed, Cochrane Library, Web of Science, Scopus and Embase). In addition, references of eligible studies were also scanned during data extraction to identify other potential studies that met inclusion criteria. Only published research articles were sought. Publications such as theses, dissertations, reviews, editorials (opinions, letters and comments) were excluded.

To develop a search strategy following the Preferred Reporting Items for Systematic Reviews and Meta-Analyses (PRISMA) statement [31] using different keywords derived from the main keywords (malaria, self-medication and sub-Saharan Africa), Boolean operators “OR” and “AND” were used to broaden chances of retrieving more relevant publications and improve search sensitivity respectively (Table 1). In addition, references of identified papers were also scanned to identify other studies medication in sub-Saharan Africa; Categorise factors fuelling malaria self-medication in the region, and design a prototype of policies that are applicable in sub-Saharan African context to limit the unnecessary use of antimalarials.

**Table 1:** List of search terms used in databases (PubMed, Scopus, Web of Science, Embase and Cochraine Library).

Search terms for ‘malaria'		Search terms for ‘selfmedication'		Search terms for ‘sub-Saharan Africa'
'Malaria’ OR ‘Malaria fever’ OR ‘Human malaria'	**AND**	'Self-medication’ OR ‘Self-medication’ OR ‘Self treatment’ OR ‘Self-prescription’ OR ‘Self-prescription’ OR ‘Home treatment'	**AND**	'Sub-Saharan Africa’ OR ‘Black Africa’ OR ‘Region of Africa South of Sahara'

The research questions were: what are the reasons for malaria self-medication in sub-Saharan Africa? What are the common drugs used for that met the inclusion criteria.

The inclusion criteria for this study were as follows: Observational studies (cross-sectional, cohort and case-control) documenting primary data on malaria self-medication; Studies must have been undertaken in sub-Saharan Africa; Studies must be written in English; No restriction in the date of publication; No restriction regarding age and gender in the study population. Exclusion criteria included: Studies on malaria self-medication outside sub-Saharan Africa; Studies on self-medication generally (with no ties to any disease); Studies on self-medication with ties to a disease other than malaria; Abstract-only papers such as proceedings; Editorials; Conferences; Studies without available full texts; Case reports; Book chapters, and Systematic review studies.

**Figure 1:**
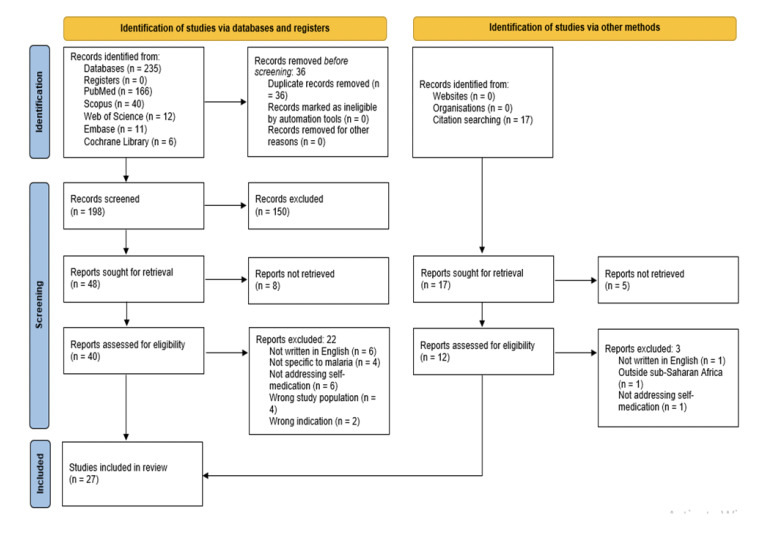
PRISMA flow diagram of the study identification and selection process.

Search of databases (PubMed, Scopus, Web of Science, Embase and Cochrane Library) identified 235 studies while manual search (other methods such as reference list search of included studies) identified 17 studies (Figure 1). Titles and abstracts of the identified studies were screened using Covidence software [32] to exclude duplicates, which were 36 in number. Titles and abstracts of the studies were screened for the inclusion criteria resulting in the exclusion of a further 150 studies. 27 studies were selected in line with the PRISMA flow diagram [31], and were deemed to have fulfilled the inclusion criteria.

Data items from eligible studies were extracted uniformly and were recorded in a table. The following items/characteristics were extracted: paper (author and year), location of study, study population, study aim/objectives, study design, sample size, self-medication prevalence, reason(s) for self-medication, medication type and source of medication.

Qualitative and quantitative data synthesis and analysis were performed. For qualitative analysis, descriptive summaries of reported reasons for self-medication, common drugs for malaria self-medication and sources of drugs for malaria self-medication were shown in a table and graphs. For quantitative analysis, forest plots of the pooled prevalence as well as subgroup analysis of prevalence and regression of logit event rate with respect to period of publication, region and country of study populations in the included studies were generated using Comprehensive Meta-Analysis (CMA) software, highlighting the overall prevalence and heterogeneity in the studies. A funnel plot was also generated to verify publication bias in the included studies.

## Results

Among the 27 included studies, there were 23 cross-sectional studies, 3 cohort studies and 1 case-control study (see Appendix). The publication dates of the studies ranged from 1989 to 2021, 93% of which were published between 2000 and 2021. The studies were from 11 out of 48 countries of sub-Saharan Africa: Nigeria (8), Tanzania (5), Ghana (2), Benin (2), Cameroon (2), Ethiopia (2), Congo DRC (2), Kenya (1), Sudan (1), Uganda (1) and Togo (1). About two-thirds of the studies focused on infants and mothers (nursing mothers, pregnant women and caregivers). Sample sizes in the included studies varied from as low as 23 in Kenya [33] to as high as 3171 in Nigeria [34].

Only 12 (44%) of the studies provided reasons for self-medication in the treatment of malaria in sub-Saharan Africa. FigureFigure 2A shows an aggregate of reasons from respondents in the twelve studies, the proportion of which is based on their frequencies in the studies. Most respondents cited high cost of hospital consultation (22%), long period of waiting in hospitals (17%), availability of cheap drugs at drug shops and informal markets (11%) and long distance to health facilities (8%) as reasons for engaging in self-medication.

**Figure 2:**
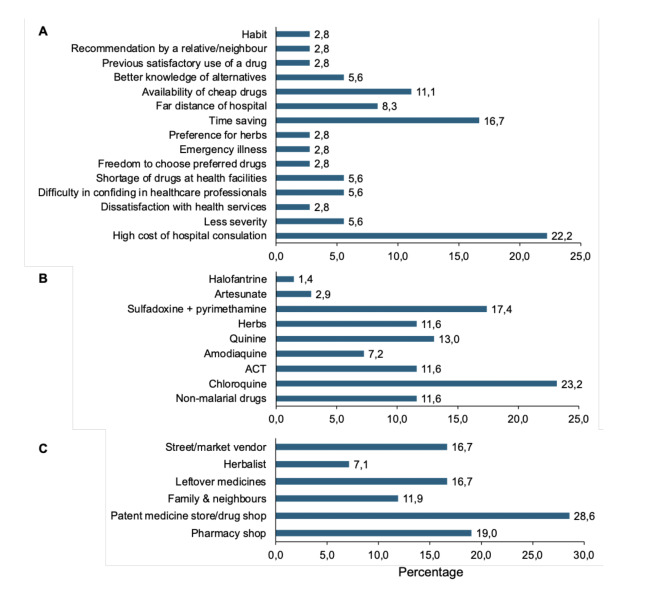
A: Reasons for malaria self-medication in sub-Saharan Africa (n=12 studies), B: Common drugs for malaria self-medication (n=24), C: Sources of drugs for malaria self-medication (n=17).

Twenty-four studies (89%) provided information from the respondents on the common drugs used in the self-treatment of malaria, the proportion of which is based on their frequencies in the studies. Most respondents indicated the use of chloroquine (23%), sulfadoxine and pyrimethamine (17%), and quinine (13%), with ACT, herbs and non-antimalarial agents (other antimicrobials, antipyretics, analgesics and supplements) trailing at 12% each (Figure 2B).

Information from respondents on the common sources of drugs for self-treatment of malaria was provided by 17 studies (63%), the proportion of which is based on their frequencies in the studies. Most indicated patent medicine store/drug shop (29%) or pharmacy store (19%) as common sources (Figure2C). Leftover medicines from previous self-treatments or previous visits to clinics and drug vendors in the streets and markets are also a substantial source of drugs for self-medication, accounting for about 17% each.

The prevalence of self-medication with antimalarials in the included studies ranged from 17.8% in Ethiopia [35] to 83% in Togo [36]. The pooled prevalence of malaria self-medication in the included studies is 55.3% (95% CI: 48.6 – 61.8) as determined in the CMA software [37] (FigureFigure 3).

**Figure 3:**
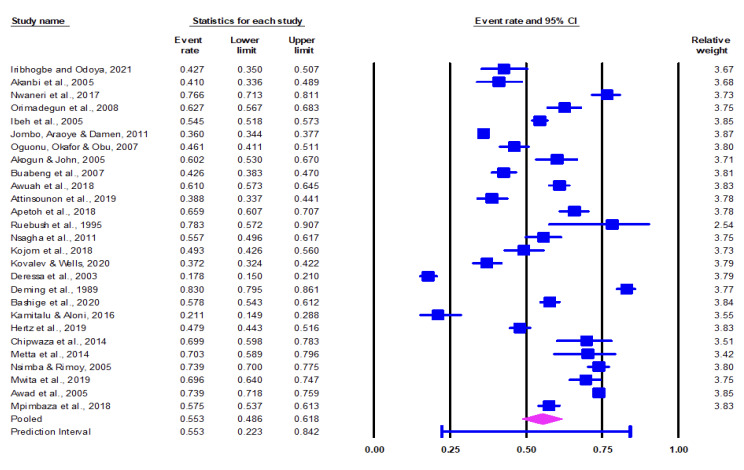
Forest plot of pooled prevalence (95% CI) of malaria self-medication in sub-Saharan Africa.

There was a high heterogeneity among the included studies with Q-value at 1469.536 (df=26) and I^2^ statistic at 98% (p<0.001), suggesting that the variance in observed effects reflects variance in true effects rather than sampling error. Even though the mean effect size was 0.553 (55.3%), the true effect size at 95% CI of comparable populations fell between the intervals of 0.22 (22%) to 0.84 (84%).

**Figure 4:**
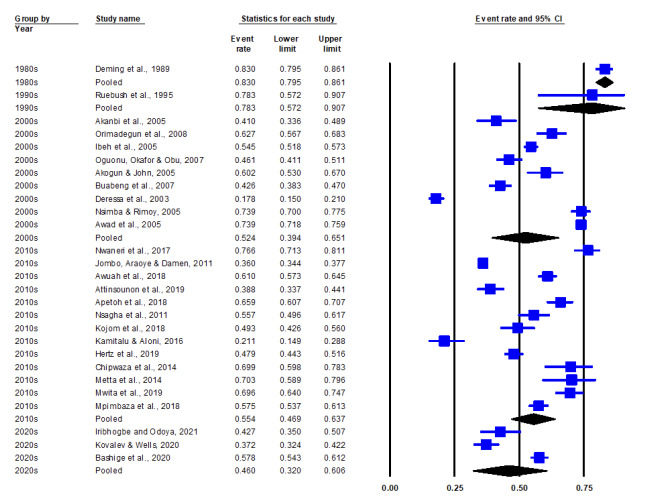
Sub-group analysis by period of the prevalence of malaria self-medication in sub-Saharan Africa.

**Figure 5:**
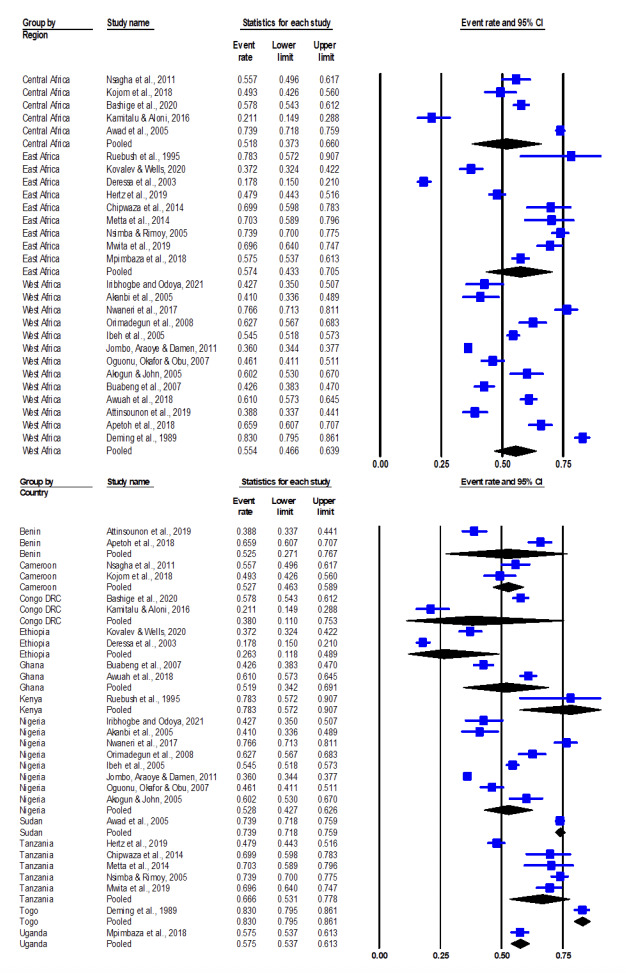
Sub-group analysis by region (top) and country (bottom) of the prevalence of malaria self-medication in sub-Saharan Africa.

The 27 included studies span across 34 years (from 1989 to 2021). The trends in prevalence of malaria self-medication across covariates of period, region and country are shown in Figure 4 and Figure 5, respectively. The identified periods of 1980s, 1990s, 2000s, 2010s and 2020s had pooled prevalence values of 0.83 (83%), 0.783 (78.3%), 0.524 (52.4%), 0.554 (55.4%) and 0.460 (46%) respectively (Figure 4). Pooled prevalence values by region varied from 0.518 (51.8%) in Central Africa, 0.554 (55.4%) in West Africa to 0.574 (57.4%) in East Africa (Figure 5). Pooled prevalence values by country are as follows: Ethiopia (26.3%), Nigeria (44.7%), Benin (52.3%), Cameroon (52.8%), Ghana (53.3%), Congo DRC (53.7%), Uganda (57.5%), Tanzania (61.1%), Sudan (73.9%), Kenya (78.3%), Togo (83%) (Figure 5).

**Figure 6:**
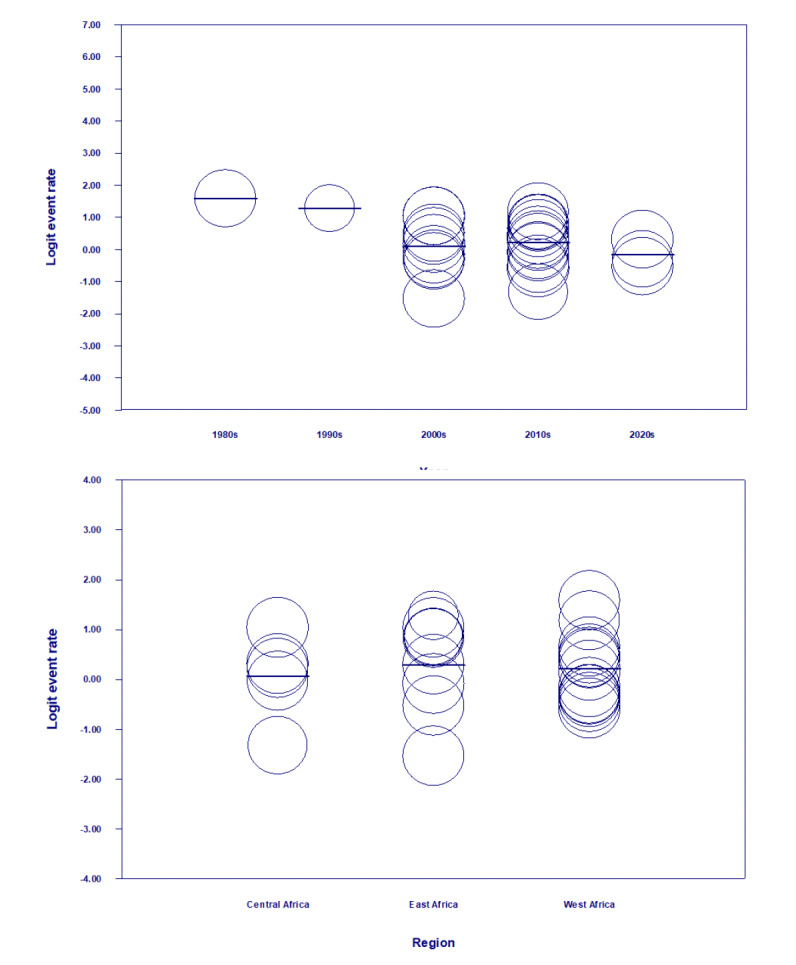
Regression of Logit Event on period (top) and region (bottom).

Random effects model meta-regression analysis shows that event rates (prevalence) somewhat decreased from the 1980s (83%) to the 1990s (78.3%), 2000s (52.4%), 2010s (55.4%) and then 2020s (46%) (FigureFigure 6), but the association between the periods is not statistically significant (p=0.1617). Also, the association between the regions is not statistically significant (p=0.8601) as the pooled prevalence was somewhat higher in East Africa (57.4%) and West Africa (55.4%) than in Central Africa (51.8%) (Figure 6).

The reported factors that influenced and fuelled malaria self-medication as identified in the included studies include: low income level [8,38-41], cheap availability of non-prescription drugs in patent medicine stores and pharmacies including informal markets (street and market vendors) [7, 42-43], previous satisfactory use of particular drugs [8,42,44], long waiting period at health facilities [7], emergency illness [7], recommendations by family members and neighbours [42], preference for herbal remedies due to perceived better efficacy [44-45], large family size [38], lack of health insurance [38-39], previous bad experience (child death) [45], long distance to health facilities [7], higher level of education [46-47], or increasing of age of mothers and care-givers [8,45,47]. However, in some studies [46,48-49] it was argued that younger mothers are more likely to self-medicate than older ones.

The respondents in all the included studies did not seek medical diagnosis to properly define the disease before medication. Some respondents based their decision for self-medication on a single symptom such as fever [38], while others based it on a combination of symptoms such as fever, headache, loss of appetite and malaise [8,33,36,47,50]. Other inappropriate practices in the use of antimalarial agents identified in this study include: incorrect dosage (under-dosing and overdosing) of medication in more than 50% of respondents [41-43,45,50-51], use of drugs (other antimicrobials, antipyretics, analgesics and supplements) other than antimalarials in malaria treatment [34,38, 41-44,46,47,50,52-53], combination of both herbal and pharmaceutical products for treatment at same time [38], change of drugs following persistence (or severity) of symptoms without advice from medical personnel [8,52], obtaining drugs from unauthorised sources such as street and market vendors, neighbours and herbalists including leftover medicines from previous treatments [35-36,40,41-45,49, 50-52,54], and shortening of the course of medication [8,35,47], with the intent of saving tablets for the next episode of malaria [35].

The included studies in this review reported both positive and negative outcomes in the aftermath of self-medication with antimalarial agents. The positive outcomes attributed to the use of antimalarials drugs included decrease in parasite density [55,56] and improvement in condition [54]. Negative outcomes included abdominal upset [40], rashes [40], weakness of the body [40], severe malaria [41,48,54,56], no improvement in condition [7], increased mortality [41] and no change in parasite count [41, 54].

## Discussion

Self-medication with antimalarials is a prevalent issue in sub-Saharan Africa, contributing to the rising cases of antimalarial drug resistance in the region [6-7]. This study aimed to synthesise evidence on the prevalence and driving factors of this practice to inform policymakers in designing effective control interventions. The findings indicate that various factors fuel self-medication, including the high cost of hospital consultations [36, 41-43,45,48,50,53,55], time-saving considerations [9, 36,^43^,^55^], availability of affordable drugs in local markets [36,43], long waiting times at health facilities [8,36], considerable distance to healthcare centers [8,36,53,55], as well as previous positive experiences with self-administered drugs [43]. These findings agree with prior studies that highlight cost and accessibility as primary drivers of self-medication [6,16]. While over 70% of the population subsist on less than US$1.90 per day, hospital consultation for malaria treatment averaged US$17.48 per patient, thereby not only becoming a major economic burden for the majority of the population but also constituting an important barrier to care-seeking [57].

The common medications used for self-treatment in the region include chloroquine, sulfadoxine, pyrimethamine, artemisinin-based combination therapies (ACTs), non-antimalarial agents like analgesics, and traditional herbs. Historically, herbs have been a source of modern antimalarial drugs such as artemisinin and quinine derivatives [58], and promising antimalarial activity has been observed in various African plants, including the neem tree (*Azadirachta indica*) and African wormwood (*Artemisia afra*) [59]. However, the misuse of these medications, characterised by shortened treatment courses, incorrect dosages (under-dosing and overdosing), and the use of non-antimalarial drugs, often leads to severe complications, as symptoms may be alleviated without addressing the underlying disease, potentially masking other conditions like typhoid [6]. This inappropriate use of antimalarials, especially under-dosing, can lead to sub-therapeutic drug levels, fostering antimalarial drug resistance and ultimately eroding the clinical effectiveness of these vital treatments [5,14].

Drugs for self-medication are primarily obtained from neighbourhood drug stores, pharmacies, street and market vendors, and even leftover medicines from previous prescriptions (see Appendix). The informal drug market thrives in sub-Saharan Africa due to its accessibility, convenience, and affordability, serving as a critical source of medication for many low-income individuals [60]. Little wonder an argument by Foster [20], about three decades ago, is still valid that self-medication with antimalarials will remain the main source of treatment for the disease for the foreseeable future since more than half of antimalarials in distribution is in the informal market, stressing that the practice should be understood and improved. This poses a significant regulatory challenge, as a substantial portion of antimalarials are distributed through this informal sector [16]. The activities of the informal drug market seem to be beyond the control of regulatory agencies, and policy makers might be having a dilemma as to what course of action to take. Efforts to regulate this market, such as training drug vendors, have faced resistance due to fears of legitimising self-medication practices [16].

The pooled prevalence of malaria self-medication in sub-Saharan Africa is estimated at 55.3%, although there are notable variations across periods and countries, ranging from 17.8% in Ethiopia to 83% in Togo. While the prevalence has shown a progressive decrease from 83% in the 1980s to 46% in the 2020s, this trend is not statistically significant. Regional differences were also observed, with pooled prevalence rates of 51.8% in Central Africa, 55.4% in West Africa, and 57.4% in East Africa, though these associations were not statistically significant. These findings underscore the need for more comprehensive data on prevalence, as previous review studies on malaria self-medication in the region have focused more on thematic issues rather than quantitative prevalence data [6,16,60].

The factors driving self-medication are essentially risk factors that stem from the structural challenges within healthcare systems in sub-Saharan Africa. In some areas, such as Tanzania's Kilombero region, the absence of public health facilities and the presence of numerous private drug stores make self-medication the only viable option for residents [44]. Drug stores often serve as the primary point of treatment due to their accessibility and affordability [62]. Addressing these underlying structural issues through strong political will is crucial for mitigating the practice of self-medication.

Inappropriate practices identified in this study include incorrect dosage (under-dosing and overdosing) of medication, use of drugs other than antimalarial agents, combination of both herbal and pharmaceutical products for same treatment, change of drugs following severity of symptoms without recourse to medical advice, obtaining drugs from unauthorised sources, and shortening of the course of treatment once symptoms subside. Cases of both under-dosing and overdosing of antimalarial agents were reported in studies carried out in Uganda, Mali and Congo [16]. There were also reports of respondents abandoning medications midway once symptoms subside, and they keep the remnants of the drugs for future episodes of the disease or give them out to family and friends [16]. Inappropriate use of antimalarials especially under-dosing provides the malaria parasite with sub-therapeutic levels of the drug, and this could lead to antimalarial drug resistance [6]. Inappropriate use of antimalarials could lead to failure of therapies, essentially eroding their clinical usefulness and paving way for antimalarial drug resistance [63].

A significant challenge in malaria self-medication is the difficulty in accurately diagnosing the disease, as many tropical illnesses share similar symptoms like fever, headache, and malaise. This often leads to self-diagnosis based on clinical signs rather than confirmed parasitic infection, as evidenced by studies where respondents based their self-medication decisions on single or multiple symptoms. This can result in misdiagnosis and delayed appropriate treatment, potentially leading to serious consequences. Individuals (especially non-pregnant adults) with clinical immunity to malaria in endemic areas may not show symptoms of the disease [64], and this could mean that not all febrile cases are due to malaria. Even some official operating procedures such as the SBET for travellers make provision for taking of antimalarial medications on incidence of fever before consulting medical personnel [25, 65], without regard to the fact that fever occurs while travelling to malaria-endemic areas due to various reasons [66]. Clinical signs such as fever do not conclusively prove the presence of malaria [65-68]. Therefore, a major deficiency in malaria self-medication practices is the lack of clinical evaluation of the condition by qualified medical personnel which could result in misdiagnosis of the disease and delay in seeking appropriate treatments [65,69], or even possible serious consequences [65]. This calls to question the applicability of portable and convenient diagnostic methods such as RDTs in malaria-endemic areas for the reach of rural dwellers and low-income earners who are distant from health facilities. While rapid diagnostic tests (RDTs) offer a portable and convenient diagnostic method, concerns remain regarding their proper usage [66] and interpretation of results [62,66], particularly in rural and low-income settings. Even though robust evidence on value-for-money for retail sector RDTs use in sub-Saharan Africa is not yet available, RDTs (based on a retail price of US$ 0.33 per test) may be more cost-effective in higher transmission settings, where a greater proportion of individuals with suspected cases reside [70]. In this review, most respondents in the studies [8,^33^,^36^,^47^,^50^] based their conclusion of having malaria on a combination of symptoms (fever, loss of appetite, weakness and headache) or even on a single symptom (fever) [38]. This result agrees with Akilimali *et al.* [6] to the effect that most studies on self-medication with antimalarial agents did not describe how the disease was defined and based treatments on symptoms such as fever and malaise. A study on multi-method assessment of patient with febrile cases by Ghai *et al.* [67] revealed that over 60% of patients would have been misdiagnosed with malaria and would have been treated with antimalarial drugs if only clinical diagnosis was employed. Even though WHO’s HMM strategy involves community health workers who somewhat provide enlightenment to caregivers on how to recognise suspected malaria cases, it can be argued that this still does not provide evidence of diagnosis that should warrant treatment. Moreover, it is argued that presumptive treatment of all febrile cases with antimalarials could speed up the development of parasite resistance [61]. Use of portable diagnostics such as RDTs could prove useful and efficient in targeting patients suspected to be suffering from malaria if adopted in the management of the disease at home [61].

Mothers, particularly nursing and expectant mothers, play a crucial role in managing childhood illnesses, especially since children <5 yrs account for a large proportion of malaria deaths in the region [4]. This highlights the significance of the role that mothers play in management of illnesses in children and how their involvement could be enhanced through a framework that enables them to make informed decisions regarding diagnosis and treatment options for febrile illnesses. Effective malaria management in under-aged children which requires mothers to seek, obtain and appropriately use medications is linked to timely decision making, accessibility and correct use of the drugs [71]. A targeted training of mothers to recognise malaria symptoms in their children and determine appropriate course of action can reduce <5 yrs mortality by about 40% [72]. Their treatment-seeking behaviours are influenced by factors like education; and lack of formal education can lead to inappropriate practices such as combining herbal and orthodox medicines [73]. Furthermore, poor adherence to guidelines regarding contraindicated antimalarial drugs during pregnancy is a concern [74, 75]. Therefore, targeted education initiatives and regulatory changes that empower mothers to make informed decisions about diagnosis and treatment are essential since they act as the first point of care for their children when they are ill [76].

The widespread practice of self-medication poses a considerable threat to malaria control and elimination efforts in sub-Saharan Africa, primarily due to its link with increasing antimalarial drug resistance. Achieving the global technical strategy for malaria 2016-2030, which aims to reduce incidence and mortality by 90% by 2030, is challenged by factors including drug resistance, weak healthcare systems, and insufficient political will [77]. Promoting the correct use of antimalarials, expanding diagnostic testing, ensuring quality-assured treatments, and strengthening regulatory frameworks to remove inappropriate drugs from informal markets are critical steps to combat drug resistance and accelerate progress toward malaria elimination [78].

### Limitations

One of the limitations of this review is unequal distribution and territorial coverage of included studies. The 27 included studies only came from 11 out of 48 countries that make up sub-Saharan Africa. There are four regions in sub-Saharan Africa: Central Africa, East Africa, West Africa and Southern Africa, with no study from the latter. About 52% of the included studies came from West Africa alone. This may not be representative enough of the true situation of malaria self-medication in sub-Saharan Africa.

Only studies published in English were included. Sub-Saharan Africa is home to countries with other official languages other than English such as French and Portuguese. Therefore, studies on malaria self-medication which might have been published in these languages were automatically excluded.

Being the only reviewer could be another limitation of the study and this might have introduced some kind of bias in selecting included studies while applying the inclusion and exclusion criteria. There are also some methodological limitations. None of the included studies provided any record of the recall period, thereby introducing some kind of recall bias. Also, self-reported tools such as questionnaire used in most studies have a high potential for social desirability bias which is a participant’s tendency to provide socially acceptable responses rather than choosing responses that reflect the true nature of their practices.

### Recommendations

There is need for more studies on malaria self-medication in sub-Saharan Africa, especially in Southern Africa and across more countries. The suggestion by Foster [20] that medication with antimalarials be understood and improved should be critically examined by policy makers in sub-Saharan Africa. The problem of poverty is a huge factor fuelling self-medication in the region, and as long as majority of the population are low-income earners, they would always prefer cheaper alternatives of treatment. In order to ensure that treatment is available as widely as possible, WHO’s HMM should be fully implemented in areas with poor access to formal health services as many people who need treatment are not served [18]. The HMM strategy should not only be limited to the involvement of community health workers in providing home treatment of malaria cases; it should also involve pharmacy owners and patent medicine dealers, incorporating training sessions on antimicrobial stewardship, correct use of antimalarials, antimalarial drug resistance and implications of inappropriate practices in malaria self-medication.

## Conclusions

Prevalence of malaria self-medication in sub-Saharan Africa is high with a pooled prevalence of 55.3%. The driving factors of the practice comprise poverty, cheap availability of non-prescription drugs in the informal market, long waiting period at health facilities, long distance to health facilities, preference for herbal remedies due to perceived better efficacy, large family size, or lack of health insurance. This presents a significant dilemma for policy makers who, on the one hand, would find it difficult to mount a fight against the practice through regulatory mechanisms due to prevailing poverty in the region, or on the other hand, fear that encouraging it openly would not only legitimise the practice but also perpetuate it. The latter would not only complicate the problem of antimalarial drug resistance but also worsen the mess of drug distribution in the region through the legitimisation of the activities of the informal market. Both scenarios are a nightmare and do not offer any sustainably feasible pathway for a clean practice of malaria management.

It is hoped that decision-makers would stop being indifferent to the practice of malaria self-medication in sub-Saharan Africa and step in to provide some guidance to all the stakeholders on the way forward because antimalarial drug resistance is a reality, and self-medication with antimalarials has been implicated by several studies to be part of the cause of antimalarial drug resistance which poses an enormous threat to decades of advances in malaria control and has the potential to put millions of lives at risk of malaria in sub-Saharan Africa.

## Appendix

**Appendix 1.** Characteristics of the studies included in the analysis.

S/N	Paper (Author(s) and year)	Country	Study population	Study aim	Study design	Sample size	Prevalence of self-medication (%)	Reason(s) for self-medication	Me dication/drug type	Source of medication
1	Iribhogbe and Odoya (2021)	Nigeria	Post-partum mothers	To investigate the practice of self-medication and its determinants and identify potential areas of public health intervention for mitigating the risk of the practice for both mother and child.	Cross-sectional	150	(for themselves) (for their newborns)	Treatment is expensive, preference for herbal medicine	ACT, herbal medicine, chloroquine, quinine	Patent medicine vendors, left over medicine
2	Akanbi et al. (2005)	Nigeria	Pregnant women	To investigate the effect of chloroquine and pyrimethamine self-medication on malaria infection and anaemia in pregnancy prior to attending an antenatal clinic	Cross-sectional	156	41	Not given	Chloroquine, pyrimethamine	Not given
3	Nwaneri et al. (2017)	Nigeria	Under-five children	To document and identify the impact of home-based management of malaria practices on malaria prevalence, level of parasitaemia and mortality in under-five children in a tertiary health facility in south-south region of Nigeria	Cross-sectional	290	76.6	High cost of treatment	ACT, paracetamol, chloroquine, artesunate, and sulfadoxine + pyrimethamine (SP), quinine, camoquine, halofantrine	Patent medicine vendors, clinics (from previous visits), neighbours
4	Orimadegun et al. (2008)	Nigeria	Children (6-59 months)	To evaluate the effects of prehospitalization use of antimalaria drugs on the morbidity and outcome of severe malaria cases in children admitted to paediatric hospitals in Ibadan, Nigeria	Cross-sectional	268	62.7	Not given	Chloroquine, Amodiaquine, Sulphadoxine-pyrimethamine	Not given
5	Ibeh et al. (2005)	Nigeria	Nursing mothers	To assess the pattern of home treatment of malaria in under-fives	Cross-sectional	1260	54.5	Not given	Chloroquine, and pyrimethamine/s ulfadoxine	Not given
6	Jombo, Araoye & Damen (2011)	Nigeria	Adult women	To assess people's the practice of self-medication and choice of drugs for treatment malaria	Cross-sectional	3171	36	Not given	Chloroquine, Panadol, septrin	Not given
7	Oguonu, Okafor & Obu (2007)	Nigeria	Caregivers of children aged 6-59 months	To assess the knowledge and attitude of caregivers in malaria treatment of children in rural and urban areas	Cross-sectional	382	46	Not given	chloroquine, sulphadoxine-pyrimethamine	Patent medicine shop, herbalist
8	Akogun & John (2005)	Nigeria	Nursing mothers	To assess the knowledge of nursing mothers in the home management of malaria	Cross-sectional	186	60.2	Not given	Herbs, antipyretics	Herbalist, medicine shop
9	Buabeng et at. (2007)	Ghana	Children and adults	To evaluate the effectiveness and quality of home-based and health facility-based management of malaria in two Ghanian communities	Cohort study	500	43	Not given	chloroquine, sulphadoxine-pyrimethamine, herbal preparations and amodiaquine	licensed chemical sellers, pharmacies, neighbouring clinics, left-over medicines at home
10	Awuah et at. (2018)	Ghana	Individuals of reproductive age (men: 15-59; women: 15-49)	To examine factors associated with seeking alternative treatment as the first response to malaria	Cross-sectional	707	61	Not given	Not given	Not given
11	Apetoh et at. (2018)	Benin	Children (12 years and below)	To describe and document the home management of malaria among caregivers, pharmaceutical products used and the factors associated with the practice.	Cross-sectional	340	66	Not given	Herbal medicine, paracetamol, quinine, eferalgan, amoxicillin, and ACT	Not given
12	Attinsounon et at. (2019)	Benin	Adults	To study the practice of antimalarial self-medication and identify associated factors	Cross-sectional	335	38.8	High cost of consultation fee, lack of time to go for consultation , good knowledge of one's illness, previous satisfactory use of the drug, recommend ation by a relative or friend, easy access to medicines in pharmacies and informal market	Quinine, artemisinin-based combination therapy, sulfadoxine-pyrimethamine, antipyretics/anal gesics.	Pharmacy, informal street market, remaining old stocks, supply by family/friend
13	Ruebush et at. (1995)	Kenya	Mothers (18-45)	To assess the level of knowledge of malaria and antimalarial therapy and how residents deal with febrile illnesses locally	Cohort	23	76.9	Not given	Chloroquine, malaraquin	Not given
14	Nsagha et at. (2011)	Cameroon	Adults (men and women)	To evaluate people's knowledge and practices relating to the choice and source of antimalarials, self-medication, malaria dosage and resistance	Cross-sectional	253	55.7	Relaxed rule on drug acquisition	Chloroquine, nivaquine, analgesics, panadol, paracetamol, native drugs	Health centre, shop, hawkers, open market, herbalist
15	Kojom et at. (2018)	Cameroon	Mothers of under-five	To assess the prevalence, patterns and predictors of antimalarial self-medication	Cross-sectional	213	49.3	Habit, lack of money and lightness of symptoms	Chloroquine	Pharmacy and street medicine stores
16	Kovalev and Wells (2020)	Ethiopia	Household caregivers	To assess the types of selfinterventions undertaken by individuals to treat perceived symptoms of malaria in a family-member prior to or instead of seeking care at a medical centre	Cross-sectional	366	37.3	High cost of modern medicine, far distance of health facilities, time-saving, low severity of illness	Chloroquine, herbs, and artemether and lumefantrine, quinine	Patent medicine store, past prescription leftover, market, neighbours
17	Deressa et al. (2003)	Ethiopia	Heads of households	To assess how communities manage malaria (sources and types of antimalarial drugs used, duration of antimalarial treatment, determinants of when healthcare is sought) to help in the design of malaria control strategies and antimalarial drug policy.	Cross-sectional	630	17.8	Closeness to him, cheap drugs, easily accessible and waiting time is short, dissatisfacti on with health sendees	Chloroquine, sulfadoxine-pyrimethamine, primaquine	Malaria control programme, drug shop, market, private clinic, health station
18	Deming et al. (1989)	Togo	Children (5 years and below)	To determine the frequency and sources of antimalarial drug treatment as well as the adequacy of treatment administered at home.	Cohort	507	83	Not given	Chloroquine (nivaquine, amodiaquine, quinine, sulfadoxine and pyrimethamine), traditional medicine	Street vendor, market vendor, pharmacy shop
19	Bashige et ah (2020)	Congo DRC	Adults aged 15 and above	To describe the practice and frequency of self-medication during malaria treatment	Cross-sectional	785	57.8	Timesaving, difficulty in confiding in healthcare professional s, knowledge of more effective remedies	Quinine, artemether and lumefantrine, Amodiaquine Sc Artesunate, sulfadoxine and pyrimethamine	Not given
20	Kamitalu and Aloni, (2016)	Congo DRC	University students	To assess the practice of self-medication against malaria among students	Cross-sectional	133	21.1	Not given	Artemether and lumefantrine, quinine, sulfadoxine and pyrimethamine	Not given
21	Hertz et at. (2019)	Tanzania	Adults	To evaluate the prevalence of self-medication with antimicrobials in areas of low malaria transmission as well as identify risk factors for the practice	Cross-sectional	718	47.9	Not given	Analgesic, antimalarial, antihelmintic, antibacterial, herbal mixture	Not given
22	Chipwaza et al. (2014)	Tanzania	Caregivers of children (aged less than 10) and health workers	To assess self-medication practice among community members	Cross-sectional	93	70 (approx)	Drug shortage at health facilities, long waiting time at and distance to health facilities, freedom to choose preferred drugs, inability to pay hospital charges	Sulfadoxine and pyrimethamine, quinine,	Pharmacy shop, drug shop, leftover medicine at home and from neighbours/relatives
23	Metta et al. (2014)	Tanzania	Adults	To evaluate malaria self-care practices and motives for choice of drug store sendees	Cross-sectional	74	70 (approx)	Poor patient-provider relationship, unavailabili ty of medicines and high cost of treatment	ACT, amodaquine, herbal product	Drug shop, leftover medicines
24	Nsimba & Rimoy, (2005)	Tanzania	Mothers of children aged five and below	To assess the extent of malaria self-medication with chloroquine by mothers/guardians of children aged five and below	Cross-sectional	529	74	Not given	Chloroquine, analgesics (aspirin, paracetamol)	Pharmacy shop, drug shop
25	Mwita et al. (2019)	Tanzania	Caregivers of people aged 6 and above	To evaluate the magnitude and factors associated with the practice of self-medication with anti-malarials among residents	Cross-sectional	280	69.6	Emergency illness, long waiting time in the health facility, close proximity to pharmacy shop, long distance to health facility	Not given	Pharmacy shop
26	Awad et al. (2005)	Sudan	Adults	To estimate the prevalence of self-medication with antimalarials as well as evaluate factors associated with the practice	Cross-sectional	1750	73.9	Financial constraints	Chloroquine	Pharmacy shop, Street/market vendor
27	Mpimbaza et al. (2018)	Uganda	Children	To describe caregivers’ responses to seek care for children with malaria	Case-control	650	49.5	Not given	Not given	Drug shop
